# Dissected subgroups predict the risk of recurrence of stage II colorectal cancer and select rational treatment

**DOI:** 10.3389/fimmu.2023.1103741

**Published:** 2023-03-23

**Authors:** Fulong Wang, Shixun Lu, Xin Zhou, Xiaotang Di, Rujia Wu, Gong Chen, Sun Tian

**Affiliations:** ^1^ Department of Colorectal Surgery, Sun Yat-sen University Cancer Center, State Key Laboratory of Oncology in South China, Collaborative Innovation Center for Cancer Medicine, Guangzhou, Guangdong, China; ^2^ Department of Pathology, Sun Yat-sen University Cancer Center, State Key Laboratory of Oncology in South China, Collaborative Innovation Center for Cancer Medicine, Guangzhou, Guangdong, China; ^3^ Yichang Central People’s Hospital, The First College of Clinical Medical Science, China Three Gorges University, Yichang, Hubei, China; ^4^ Department of Cell Biology, School of Life Sciences, Central South University, Changsha, Hunan, China; ^5^ Carbon Logic Biotech Ltd., Foshan, Guangdong, China

**Keywords:** stage II colorectal cancer, prognosis prediction, formalin-fixed paraffin-embedded samples (FFPE samples), personalized immunotherapy, personalized adjuvant drugs

## Abstract

**Background:**

Stage II colorectal cancer(CRC) patients after surgery alone have a five-year survival rate of ~60-80%; the incremental benefit of adjuvant chemotherapy is <5%. Predicting risk of recurrence and selecting effective personalized adjuvant drugs for stage II CRC using formalin-fixed, paraffin-embedded(FFPE) samples is a major challenge.

**Methods:**

1319 stage II CRC patients who enrolled in 2011-2019 at Sun Yat-sen University Cancer Center were screened. RNAseq data of FFPE tumor samples of 222 stage II microsatellite stable(MSS) CRC patients(recurrence (n=47), norecurrence (n=175), median follow-up=41 months) were used to develop a method TFunctionalProg for dissecting heterogeneous subgroups of recurrence and predicting risk of recurrence.

**Results:**

TFunctionalProg showed significant predictive values in 222 stage II MSS CRCs. The TFunctionalProg low-risk group had significantly better recurrence free survival (validation set: HR=4.78, p-value=1e-4, low-risk group three-year recurrence free survival=92.6%, high-risk group three-year recurrence free survival=59.7%). TFunctionalProg dissected two subgroups of transition states of stage II MSS CRCs at a high risk of recurrence; each state displays distinct levels of hybrid epithelial-mesenchymal traits, CD8+ T cell suppression mechanisms and FOLFOX resistance. Based on mechanisms in two subgroups, TFunctionalProg proposed personalized rational adjuvant drug combinations of immunotherapy, chemotherapy and repurposed CNS drugs. TFunctionalProg provides different utilities from ctDNA-based prognostic biomarkers.

**Conclusion:**

TFunctionalProg was validated using FFPE samples to predict the risk of recurrence and propose rational adjuvant drug combinations for stage II CRC.

## 1 Introduction

Approximately one third of new cases of colorectal cancer(CRC) are diagnosed with stage II disease. Patients with stage II CRC after surgical resection alone have a five-year survival rate of about 60-80% (1). The incremental benefit of adjuvant chemotherapy for stage II CRC is less than 5% (2). One major challenge is the development of molecular diagnostics that can utilize formalin-fixed, paraffin-embedded(FFPE) samples to predict the risk of recurrence of stage II CRC and select effective personalized adjuvant drugs for stage II CRC patients who have a high risk of recurrence. Yet to date, reported successful development of prognostic methods of stage II CRC in FFPE samples are limited. Moreover, current prognostic methods of stage II CRC tend to adopt a simple assumption that all stage II CRC patients with recurrence are a single homogenous group. This overly-simplified disease model does not capture the heterogeneous nature of mechanisms that contribute to recurrence of stage II CRC, thus can not sensitively capture the risk of recurrence and propose effective personalized adjuvant drugs to treat high-risk stage II CRC patients.

Accumulated evidence has shown that stage II CRC patients with recurrence contain heterogeneous subgroups. The first evidence is CRC molecular subtypes. The tumor cells of the epithelial-like subtype and the mesenchymal-like subtype have almost opposite characters, and the tumor microenvironment of the majority of microsatellite instable subtype (MSI) and microsatellite stable (MSS) subtypes are distinct, yet all subtypes contain CRC that developed a recurrence (3, 4). Secondly, our previous studies showed that the existence of heterogeneous subgroups with distinct gene expression patterns is a repeated phenotype in drug resistance to targeted therapy (5), FOLFOX chemotherapy (6), and immunotherapy in CRC (7).

Therefore, if the evidence indicates that high-risk stage II CRCs contain heterogeneous subgroups, several questions remain: What are these subgroups? How do the function of these subgroups contribute to recurrence? Should different subgroups of high-risk stage II CRC patients be treated with different adjuvant drugs? What types of signals of these subgroups are preserved in FFPE samples, and how do we find them?

In this report, the clinical data of 1319 stage II Chinese CRC patients enrolled at Sun Yat-sen University Cancer Center in 2011-2019 were screened; the FFPE samples of 222 stage II MSS patients with complete follow-up and treatment data were used to generate RNAseq data. We then developed and validated a novel method TFunctionalProg to dissect subgroups of two different transition states of recurrence and to predict the risk of recurrence of stage II CRC. Based on distinct tumor characters and tumor microenvironment of two different transition states of high-risk stage II CRC, TFunctionalProg proposes personalized rational adjuvant drug combinations of immunotherapy, chemotherapy and repurposed CNS drugs.

## 2 Materials and methods

### 2.1 Stage II colorectal cancer patients

Clinical data and follow-up data from 1319 stage II colorectal cancer patients who enrolled in 2011-2019 at Sun Yat-sen University Cancer Center were screened. The main endpoint was recurrence-free survival(RFS). Time to recurrence was calculated from the date of surgery to the date of recurrence confirmed by radiological assessment. Patients who developed a recurrence within 5 years were defined as the recurrence group. To remove the potential effect of residual disease at the resection margin, patients with time to recurrence less than 2 months were excluded from this study. Patients who did not develop a recurrence were defined as the norecurrence group. To remove the potential effect of treatment, patients who received neoadjuvant or adjuvant chemotherapy/radiotherapy were excluded from the norecurrence group. The MSI/MSS status of patients was determined by immunohistochemistry for four mismatch repair proteins (*MLH1, MSH2, MSH6, PMS2*). Loss of any of these four proteins was defined as MSI, and the presence of all four proteins was defined as MSS. Because MSI patients have distinct gene expression pattern, and approximately 90% of stage II MSI patients are cured by surgery without any adjuvant chemotherapy, and current guidelines also do not recommend adjuvant chemotherapy for stage II MSI patients, only patients with MSS status were enrolled in this study (1, 8, 9). In total, the recurrence group included 47 patients and the norecurrence group included 175 patients (recurrence (n=47), norecurrence (n=175), median follow-up=41 months). The flow chart of the patient selection was summarized in **Supplementary Figure S1**. The patient characteristics are described in **Table 1**. Patients or the public were not involved in the design, or conduct, or reporting, or dissemination plans of our research.

**Table 1:** Main clinicopathological characteristics of formalin-fixed, paraffin-embedded samples of 222 stage II MSS CRC patients.

Clinical Characteristic	Training Set (n = 112) n(%)	Validation Set (n = 110) n(%)
Sex		
Male	61(54.5)	65(59.1)
Female	51(45.5)	45(40.9)
Median age, years	62	61
Median follow-up(months)	41.3	41.2
Localization		
Left	47(42.0)	58(52.7)
Right	29(25.9)	27(24.5)
Rectum	36(32.1)	25(22.7)
T		
3	79(70.5)	81(73.6)
4	33(29.5)	29(26.4)
MSS/MSI		
MSS	112(100)	110(100)
MSI	0(0)	0(0)
Recurrence developed during the follow-up period		
No	88(78.6)	87(79.1)
Yes	24(21.4)	23(20.9)
Chemotherapy		
No	97(86.6)	94(85.5)
Yes	14(12.5)	15(13.6)
Unknown	1(0.9)	1(0.9)

### 2.2 RNAseq data

Tumor slides were prepared from FFPE tumor samples using standard procedures in the pathology laboratory at Sun Yat-sen University Cancer Center. Ten FFPE slides were prepared for each tumor sample. All samples contained at least 25% tumor cells. RNA isolation and sequencing were performed according to standard lab protocols for FFPE samples. Briefly, RNA isolation was performed using a Qiagen FFPE RNA Kit and ribosomal RNA was removed. The DV100 values of isolated RNA of all samples were greater than 40%. NEB kit E7765 (NEBNext Ultra II Directional RNA Library Prep with Sample Purification Beads) was used for library preparation. RNAseq was performed on the Illumina NovaSeq6000 150bp paired-end sequencing platform. 18G data were sequenced for each sample and Q30 values of fastq data of all samples are greater than 80%. BBMap.v38 was used to trim the adapter (ktrim=r k=21 mink=10 hdist=2) and bases with low quality (trimq<15) (10). Kallisto.v0.46.1 was used to align the trimmed reads to the Ensembl human reference transcriptome Homo_sapiens.GRCh38.v96 (11). The estimated counts of genes were summarized using the tximport package and counts were normalized using the scaledTPM method (12).

### 2.3 Development of the iterative functional prognostic model TFunctionalProg

**Figure 1:**
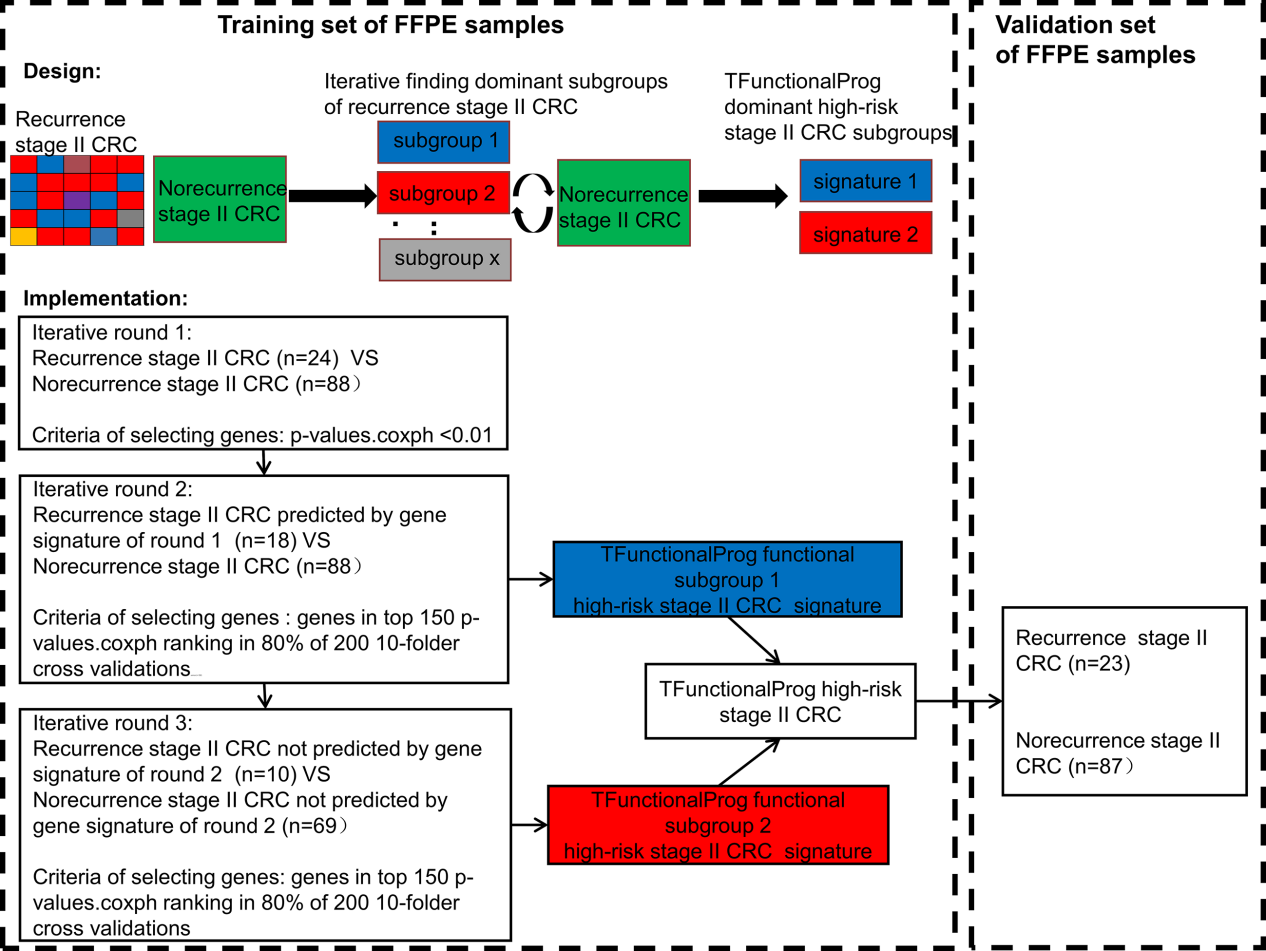
The implementation of the TFunctionalProg method dissects stage II colorectal cancer patients with recurrence into two subgroups, and then analyzes the mechanism of recurrence of the two subgroups separately.

In total, the RNAseq data of 222 stage II MSS CRC tumor FFPE samples were used to develop the TFunctionalProg method. Half were used for training (training n=112, recurrence n=24, norecurrence n=88) and half were used for independent validation (validation n=110, recurrence n =23, norecurrence n=87). **Figure 1** shows the design of the study and the implementation of the iterative workflow used for training and validating TFunctionalProg. The Cox proportional hazards regression model is used to fit gene expression values with time to recurrence data, and coxph function implemented in the R survival package was used (13). The first learning round preselected recurrence tumors with the same character, and a Cox proportional hazards regression was performed. P-values *p.coxph <*0.01 were used to select genes. The second and the third learning rounds reinforced the previous learning rounds and selected genes in the final signature. In each of these two learning rounds, a subset of recurrence samples was compared with a subset of norecurrence samples, and 200 rounds of 10-fold cross-validation were performed. In each cross-validation, the p-values of Cox proportional hazards regression *p.coxph* of genes were ranked, and genes ranked in the top 150 ranking in at least 80% of 200 cross-validations were selected as the signature. The signature was constructed using the nearest centroid model of two centroids: a highrisk centroid and a lowrisk centroid. The cutoff was optimized by the overall performance (TFunctionalProg subgroup 1: cutoff = -0.0850; TFunctionalProg subgroup 2: cutoff= -0.0005). The final model output was the binary combination of nearest centroid models of the second cross-validation and the third cross-validation. Each stage II CRC tumor sample was output into one of the two risk groups: TFunctionalProg high-risk(combined of high-risk subgroup 1 and high-risk subgroup 2) and TFunctionalProg low-risk.

### 2.4 Statistical analysis and functional analysis

Survival analysis was performed using the R package survival. The hazard ratio was calculated using the Cox proportional hazards regression model and the p-value was calculated using the log-rank test. Enriched function and biological pathway analysis were performed using FunRich v3.1.4 (14). The p-values of comparisons of individual markers and FOLFOX resistance scores between the TFunctionalProg low-risk and TFunctionalProg high-risk subgroups are calculated using the Wilcoxon rank sum test (6). To read out tumor infiltrating immune cells, we used 43 markers of 14 common tumor infiltrating immune cell types derived from TCGA data: Treg cells, Th1 cell cells, T cell cells, NK cells, NK CD56^dim^ cells, neutrophils, mast cells, macrophages, exhausted CD8 cells, DCs, cytotoxic cells, CD8 T cells, CD45 cells and B cells (15).

## 3 Results

### 3.1 Prognostic value of the TFunctionalProg model in stage II CRC tumors

The final prediction method TFunctionalProg consists of two gene sets of the iterative functional prognostic model([set 1(n=41)] **Supplementary Table S1**, [set 2(n=40)] **Supplementary Table S2**). In the training set of 112 stage II MSS CRCs (recurrence n=24, norecurrence n=88), the survival analysis resulted in a significant hazard ratio and p-value (p-value<1e-4, AUC of TFunctionalProg score=0.8764). The TFunctionalProg high-risk group contained 45.5% of samples. 100% of stage II CRC patients in the TFunctionalProg low-risk group did not develop recurrence within the follow-up period. The low-risk group had a three-year RFS rate of 100% and the high-risk group had a three-year RFS rate of 58.1% [95% CI, 45.9%-73.5%].

**Figure 2:**
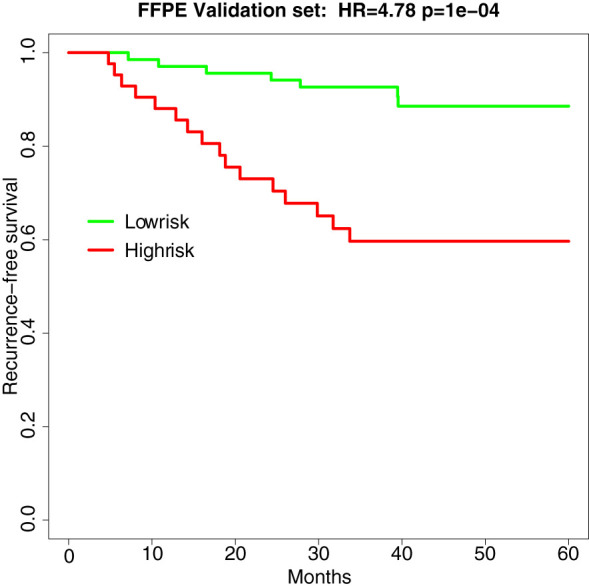
Survival curve of recurrence-free survival in the independent validation set of FFPE samples (HR=4.78, p-value=1e-4). Green is the TFunctionalProg low-risk group and red is the TFunctionalProg high-risk group. The low-risk group had a three-year recurrence-free survival rate of 92.6% and may consider forgoing adjuvant therapy. The high-risk group had a three-year recurrence-free survival rate of 59.7% and contains two subgroups. Based on the different tumor characters of two TFunctionalProg high-risk subgroups, personalized rational adjuvant chemotherapy+drug combinations may be selected.

In the independent validation set of 110 stage II MSS CRCs (recurrence n =23, norecurrence n=87), the hazard ratio and p-value of the survival analysis of the TFunctionalProg method remained significant (HR=4.78, p-value=1e-4, AUC of TFunctionalProg score=0.7001). The TFunctionalProg high-risk group contained 38.2% of samples; 89.7% of stage II CRC patients in the TFunctionalProg low-risk group did not develop a recurrence within the follow-up period. The low-risk group had a three-year RFS rate of 92.6% [95% CI, 86.6%-99.1%] and the high-risk group had a three-year RFS rate of 59.7% [95% CI, 46.1%-77.2%] (**Figure 2**).

### 3.2 Characterization of the two TFunctionalProg high-risk subgroups of stage II CRC tumors and their implications on the chemotherapy response

**Figure 3:**
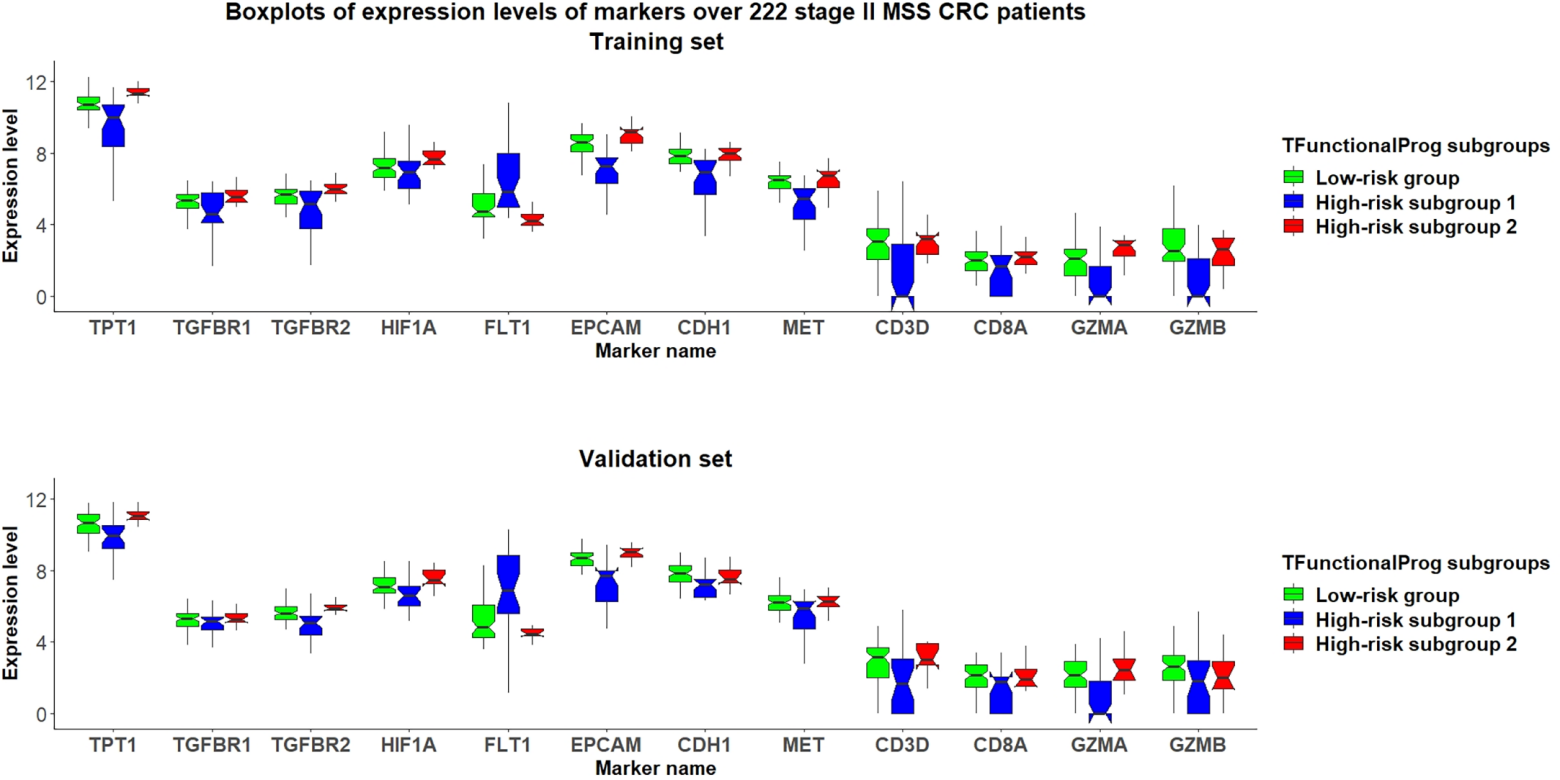
Grouped boxplots of expression levels (Y-axis) of selected markers of epithelial mesenchymal transition (*TPT1, TGFBR1, TGFBR2, FLT1*), hypoxia (*HIF1A*), epithelial phenotype (*EPCAM, CDH1, MET*) and tumor infiltrating cytotoxic T cells (*CD3D, CD8A, GZMA, GZMB*) in 222 stage II MSS CRC patients divided into three subgroups: (1) TFunctionalProg low-risk group (green), (2) TFunctionalProg high-risk subgroup 1 (blue) and (3) TFunctionalProg high-risk subgroup 2 (red). The upper panel is the training set and the lower panel is the independent validation set. The tumor characters and tumor microenvironments of the two TFunctionalProg high-risk subgroups are clearly different and suggest that recurrence of stage II CRC requires transitions from multiple distinct states.

The TFunctionalProg model identified two high-risk subgroups, both associated with a high risk of recurrence of stage II CRC. TFunctionalProg subgroup 1 [set 1(n=41)] was characterized by the downregulation of genes involved in the mitotic cell cycle *(PCNA, CDK4, RPA2, MCM6, MCM2*). In addition, ~50% of the genes in subgroup 1 have a known function in the nucleus (*PCNA, HIST1H2AH, CAV2, SETD1A, HIST1H2BK, RPA2, TNFAIP3, CACYBP, ENO1, HIF3A, UTP18, DUSP23, WT1, CDK4, SNAPC4, PIR, MCM6, MTA2, MCM2, NR2C2AP, E2F8*). The log2 scale of the mean fold changes (high-risk to low-risk) of downregulation was -0.82. Epithelial markers (*EPCAM, CDH1, EGFR, MET*) were lost in subgroup 1. Interestingly, except for the upregulation of FLT1, most classic mesenchymal markers were not found to be upregulated in subgroup 1 (blue box, **Figures 3**, **S2**, **S3**). The markers of 14 common tumor infiltrating immune cell types derived from TCGA data showed that tumors in subgroup 1 harbored considerably lower levels of markers of neutrophils (*FCGR3A*), macrophages (*CD68*), T cells (*CD3D, CD3E*), exhausted CD8+ T cells (*CD244, LAG3*), DCs (*HSD11B1*), B cells (*CD19*), CD8+ T cells (*CD8A*) and cytotoxic cells (*GZMA, GZMB, KLRD1, PRF1*) (blue box, **Figures 3**, **S4**-**S6**) **(** 15). These results showed that tumors in high-risk subgroup 1 tend to lack tumor infiltrating immune cells, begin to form a cytotoxic cell desert, and maintain a low proliferation rate.

The TFunctionalProg subgroup 2 [set 2(n=40)] showed comparable counts of tumor infiltrating immune cells, and the level of epithelial markers was maintained (red box, **Figures 3**, **S3**-**S6**). A tendency towards the upregulation of several known suppressors of anti-tumor immunity and initiators of epithelial-mesenchymal transition (EMT) was observed: *TGFBR1, TGFBR2, TGFB3* and *TPT1* (red box, **Figures 3**, **S2**). In this state, tumors in subgroup 2 maintain the upregulation of both epithelial and some mesenchymal characters (red box, **Figures 3**, **S2**, **S3**). The most significant enriched pathways in the signature of subgroup 2 could be mapped to TGF-β receptor signaling and the regulation of cytoplasmic and nuclear SMAD2/3 signaling (percentage=35.3%, p.hypergeometric=9.72e-5, **Supplementary Table S3**). Activating TGF-β pathway is an extensively documented factor of metastasis initiation in CRC (16–19). Our results are consistent with the known roles of TGF-β; however, our results also showed: (1) only ~35% of the signals of subgroup 2 can be explained by known components of TGF-β and SMAD2/3 pathway, so activating TGF-β is unlikely the only signal. Except for activating TGF-β, another observed dominant character of subgroup 2 is the upregulation of *TPT1.* (red box, **Figure 3**) (2) the high metastatic potential of CRC can be induced when TGF-β activation is mixed in tumor cells that still maintain some epithelial characters (red box, **Figures 3**, **S3**). Taken together, subgroup 2, associated with a high risk of recurrence of stage II CRC, shows characters of upregulation of anti-tumor immunity suppressors and a hybrid state of epithelial and mesenchymal phenotype.

**Figure 4:**
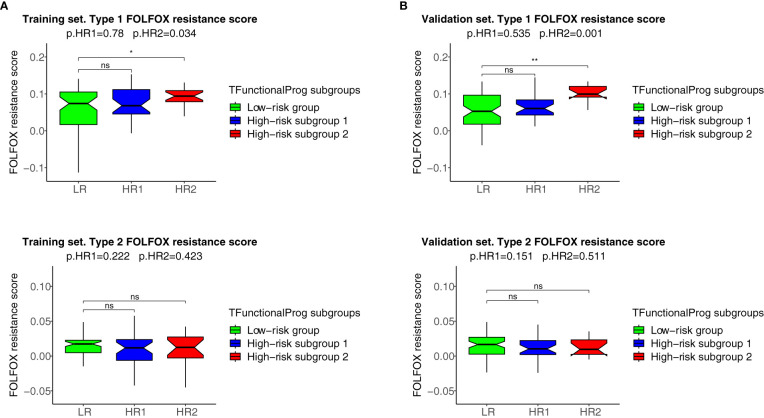
FOLFOX chemotherapy resistance scores of the TFunctionalProg low-risk group (green, LR), TFunctionalProg high-risk subgroup 1 (blue, HR1) and TFunctionalProg high-risk subgroup 2 (red, HR2). Type 1 FOLFOX resistance scores are plotted in **(1A)** (training set) and **(1B)** (validation set), and type 2 FOLFOX resistance scores are plotted in **(2A)** (training set) and **(2B)** (validation set). The significance levels of pairwise comparisons against FOLFOX resistance scores of stage II CRC patients in the TFunctionalProg low-risk subgroup (green, LR) as the reference group are shown in the figure (ns p>0.05, * p < 0.05, ** p < 0.01). In the title, the first p-value p.HR1 is the comparison between the FOLFOX resistance scores of stage II CRC patients in the TFunctionalProg high-risk subgroup 1 (blue, HR1) and the FOLFOX resistance scores of the other stage II CRC patients, and the second p-value p.HR2 is the comparison between the FOLFOX resistance scores of stage II CRC patients in the TFunctionalProg high-risk subgroup 2 (red, HR2) and the FOLFOX resistance scores of the other stage II CRC patients. Patients in the TFunctionalProg high-risk subgroup 2 (HR2) showed consistently high type 1 FOLFOX resistance scores in both the training set and validation set (p.training=0.034, p.validation=0.001). The resistance to chemotherapy in the subgroups of high-risk stage II CRC explains why adjuvant chemotherapy only showed a minimal incremental benefit in stage II CRC.

The two TFunctionalProg subgroups also showed different responses to chemotherapy. FOLFOX resistance scores were read out on all samples using the FOLFOX response signature (6). The results show that CRC patients in TFunctionalProg subgroup 2 had higher FOLFOX resistance scores (p.training=0.034, p.validation=0.001, red box, panel 1, **Figure 4**). These results partially explain why adjuvant chemotherapy has less than a 5% incremental benefit for stage II CRC patients (2). TFunctionalProg subgroup 2 high-risk patients tend to resist FOLFOX, while the tumor cells of TFunctionalProg subgroup 1 high-risk patients already lost main epithelial characters and their cytotoxic cells are depleted.

## 4 Discussion

The design of prognostic prediction methods needs to reflect the fundamental logic of biology. High-risk stage II CRC is a heterogeneous group. The underlying design of a prognostic prediction method needs to identify these distinct subgroups to capture the biology of the risk of recurrence of stage II CRC. The functions of two TFunctionalProg high-risk subgroups suggest that the recurrence of stage II CRC might not be as simple as a binary 0-to-1 transition that directly transits from tumor characters of no recurrence to tumor characters of recurrence. Rather, the development of stage II CRC recurrence might occur through multiple transition states. In our study, two stable states in CRC were identified: (1) TFunctionalProg subgroup 1 might represent a late stage that tumor cells that have already undergone EMT. Tumor cells have lost almost all epithelial characters (blue box, **Figures 3**, **S3**), have low proliferation index and an immune desert has formed (blue box, **Figures 3**, **S4**-**S6**). (2) TFunctionalProg subgroup 2 might represent an early stage of EMT where epithelial characters are still maintained by tumor cells, but the signal of EMT initiation is apparent. As a consequence, tumor cells display a hybrid state of both epithelial and mesenchymal traits. Our gene expression data in stage II CRC patients agree with the *in vivo* observation that EMT is a multi-state transition process and proceeds through intermediate transition states, in which tumor cells with hybrid states of epithelial characters and mesenchymal characters exist and can efficiently form metastasis (20–22). Both upregulation of TGF-β pathway and *TPT1* are known factors that suppress anti-tumor immunity. Activation of the TGF-β pathway can initiate the T cell exclusion phenotype and *TPT1* upregulation can induce myeloid-derived suppressor cells (23, 24). Thus, although the numbers of cytotoxic cells in subgroup 2 were not significantly decreased (red box, **Figures 3**, **S4**-**S6**), their function may be suppressed. The two transition states pose the question as to what factors drive stage II CRC to transit from the early state to the late state of recurrence. This driving factor of the transition between these two states is unclear. Interestingly, *FLT1* was upregulated in TFunctionalProg subgroup 1 (blue box, **Figures 3**, **S2**) and *HIF1A* was upregulated in TFunctionalProg subgroup 2 (red box, **Figures 3**, **S7**); these characters suggest that one driving factor might be hypoxia (25).

**Figure 5:**
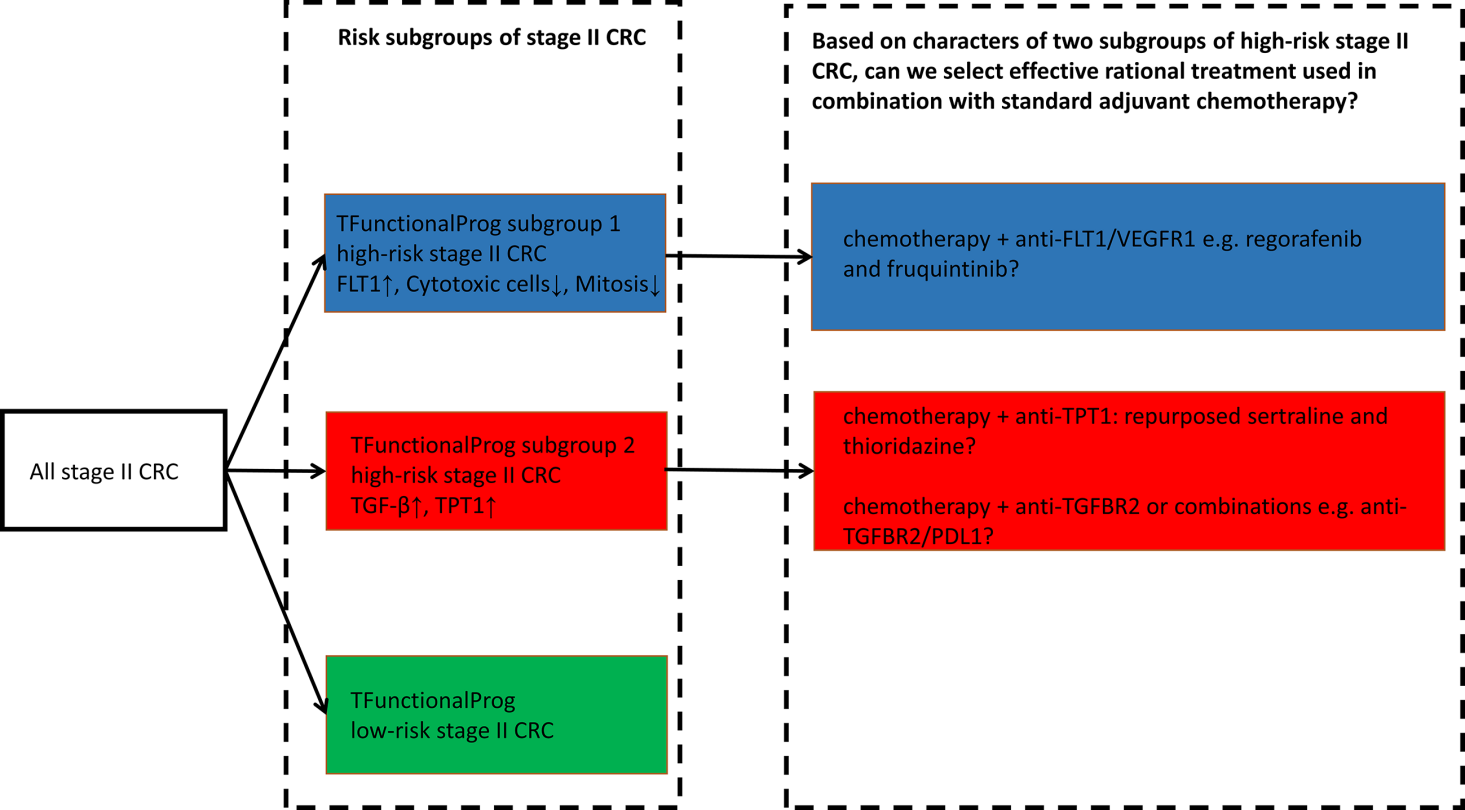
TFunctionalProg subgroups of the risk of recurrence of stage II CRC: low-risk group (green, LR), high-risk subgroup 1 (blue, HR1) and high-risk subgroup 2 (red, HR2). Different tumor characters and tumor microenvironments in TFunctionalProg high-risk subgroup 1 and TFunctionalProg high-risk subgroup 2 suggest that standard chemotherapy may be combined with different rational personalized treatment options to increase the benefit of adjuvant treatment in stage II CRC. This scheme proposes three drug combinations for further investigation.

TFunctionalProg was validated on standard FFPE slides and can be implemented as a diagnostic tool to identify stage II CRC patients at a high risk of recurrence. The TFunctionalProg low-risk group of stage II CRC has a three-year RFSrate of >92% and may consider forgoing adjuvant therapy. The TFunctionalProg high-risk group of stage II CRC has a three-year RFS rate of <60%. The current observed incremental benefit of adjuvant chemotherapy for stage II CRC patients is <5% (2). Biomarkers are already used to select stage III CRC patient subpopulations to receive personalized adjuvant chemotherapy plus immunotherapy (26, 27). It would be interesting to explore further whether different tumor characters of TFunctionalProg high-risk subgroup 1 and TFunctionalProg high-risk subgroup 2 could help select different personalized rational chemotherapy plus drug combinations to increase the benefit of adjuvant treatment for stage II CRC patients (2). Three drug combinations are proposed for further investigation (**Figure 5**):

1. TFunctionalProg high-risk subgroup 2 stage II CRC patients show a dependence on TGF-β pathway activation and *TPT1* (red box, **Figure 3**). It is important to note that the number of CD8+ T cells and cytotoxic cells is not significantly decreased in subgroup 2. Therefore, TFunctionalProg high-risk subgroup 2 CRC patients might represent a patient subpopulation that could be potentially treated by an anti-*PDL1/TGFBR2* bispecific antibody (18, 28).
2. TFunctionalProg high-risk subgroup 2 stage II CRC patients also showed upregulation of *TPT1* (red box, **Figure 3**). *TPT1/TCTP* is a switch regulating tumor reversion and loss of TPT1 results in loss of malignancy of tumor (29). Two central nervous system (CNS) drugs, sertraline and thioridazine, directly bind to *TPT1/TCTP* and inhibit its function (30). In human CRC cell lines, sertraline and thioridazine, alone or in combination with 5-FU, showed significant inhibition of tumor growth (31). A biomarker-driven drug repurposing of CNS drugs to treat TFunctionalProg high-risk subgroup 2 stage II CRC patients may be further explored.
3. TFunctionalProg high-risk subgroup 1 stage II CRC patients showed depletion of CD8+ T cells and cytotoxic cells (blue box, **Figure 3**) and upregulation of *FLT1* (blue box, **Figure 3**). *FLT1/VEGFR1* is a vascular endothelial growth factor receptor and its ligands include *VEGFA, VEGFB* and *PlGF*. The anti-*VEGFA* drug bevacizumab is a standard treatment for metastatic CRC and is associated with a benefit in terms of overall survival. Anti-*FLT1/VEGFR1* drugs that are already used in CRC include aflibercept and tyrosine kinase inhibitors such as regorafenib and fruquintinib. Patients with refractory metastatic CRC treated with regorafenib or fruquintinib showed better overall survival (32). Whether stage II CRC patients benefit from regorafenib or fruquintinib in the adjuvant setting is unknown, but the character of TFunctionalProg high-risk subgroup 1 stage II CRC patients may represent a rational subpopulation in which to start clinical studies.

A rapidly adapted biomarker for predicting stage II CRC at a high risk of recurrence is postoperative circulating tumor DNA(ctDNA) from blood samples (33). Postoperative positive ctDNA status is an indicator of minimal residual disease and is associated with a high risk of recurrence. Yet TFunctionalProg offers several different features. Firstly, the sensitivity of TFunctionalProg of predicting the recurrence of stage II CRC is between 70% (independent validation) and 85% (leave one out cross-validation), and this sensitivity is higher than the sensitivity of postoperative ctDNA-based biomarkers of predicting the recurrence of stage II CRC(estimated number of recurrence detected in ctDNA-positive group/total number of recurrence) estimated from three recent clinical trials of ctDNA-based biomarkers (34–36). It would be interesting to investigate whether circulating tumor cells of ctDNA-positive stage II CRCs correlates with a specific TFunctionalProg high-risk subgroup, and this may help the understanding of the functionality and the origin of heterogenous circulating tumor cells (37). Secondly, TFunctionalProg aims to dissect underlying biology of the recurrence of stage II CRC, thus TFunctionalProg provides biology that explains chemotherapy resistance and proposes alternative rational adjuvant treatment options. Thirdly, TFunctionalProg utilize standard FFPE slides that can be stored at the room temperature and the operational cost as molecular diagnostics is significantly lower than ctDNA-based assays. Taken together, TFunctionalProg provides a different modality from ctDNA-based biomarkers for the management of stage II CRC.

To conclude, TFunctionalProg was validated on standard FFPE slides to predict the risk of recurrence of stage II CRC. Considering high-risk stage II CRC patients, TFunctionalProg integrated functions that dissect two different subgroups of transition states of recurrence and proposed different personalized rational adjuvant drug combinations of chemotherapy, immunotherapy and repurposed CNS drugs.

## Data availability statement

The datasets presented in this article are not readily available because their access is restricted by local rules of Regulations on management of human genetic resources. Requests to access the datasets requires material transfer agreement and should be directed to wu.rujia@carbonlogicdx.com.

## Ethics statement

The studies involving human participants were reviewed and approved by Medical Ethical Committee of Sun Yat-sen University Cancer Center. The ethics committee waived the requirement of written informed consent for participation.

## Author contributions

FW: patients, collection and/or assembly of data, data analysis and interpretation, manuscript writing first draft, final approval of manuscript. SL: patients, collection and/or assembly of data, final approval of manuscript. XZ: collection and/or assembly of data, final approval of manuscript. XD: data analysis and interpretation, final approval of manuscript. RW: collection and/or assembly of data, data analysis and interpretation, final approval of manuscript. GC: concept/design, patients, collection and/or assembly of data, final approval of manuscript. ST: concept/design, data analysis and interpretation, manuscript writing first draft, final approval of manuscript.
