# Supplementary material for: Dissected subgroups predict the risk of recurrence of stage II colorectal cancer and select rational treatment
Source: Front Immunol. 2023 Mar 23;14:1103741. doi: 10.3389/fimmu.2023.1103741 (PMC10076777; doi:10.3389/fimmu.2023.1103741)

Figure S1. Flow chart of the patient selection

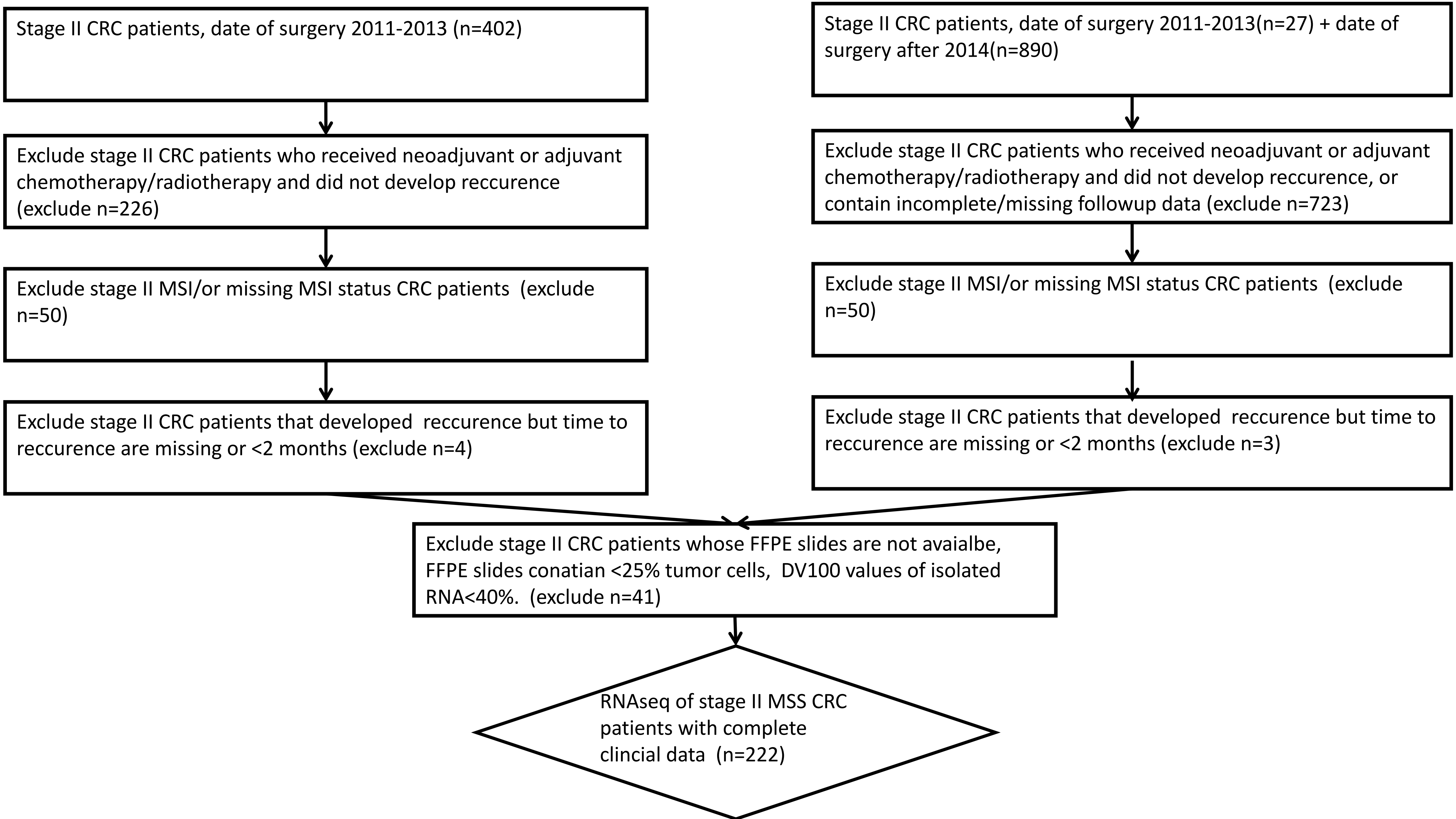

Figure S2. Boxplots of expression levels of markers of mesenchymal phenotype and epithelial-mesenchymal transition over 222 stage II CRC patients divided into three subgroups

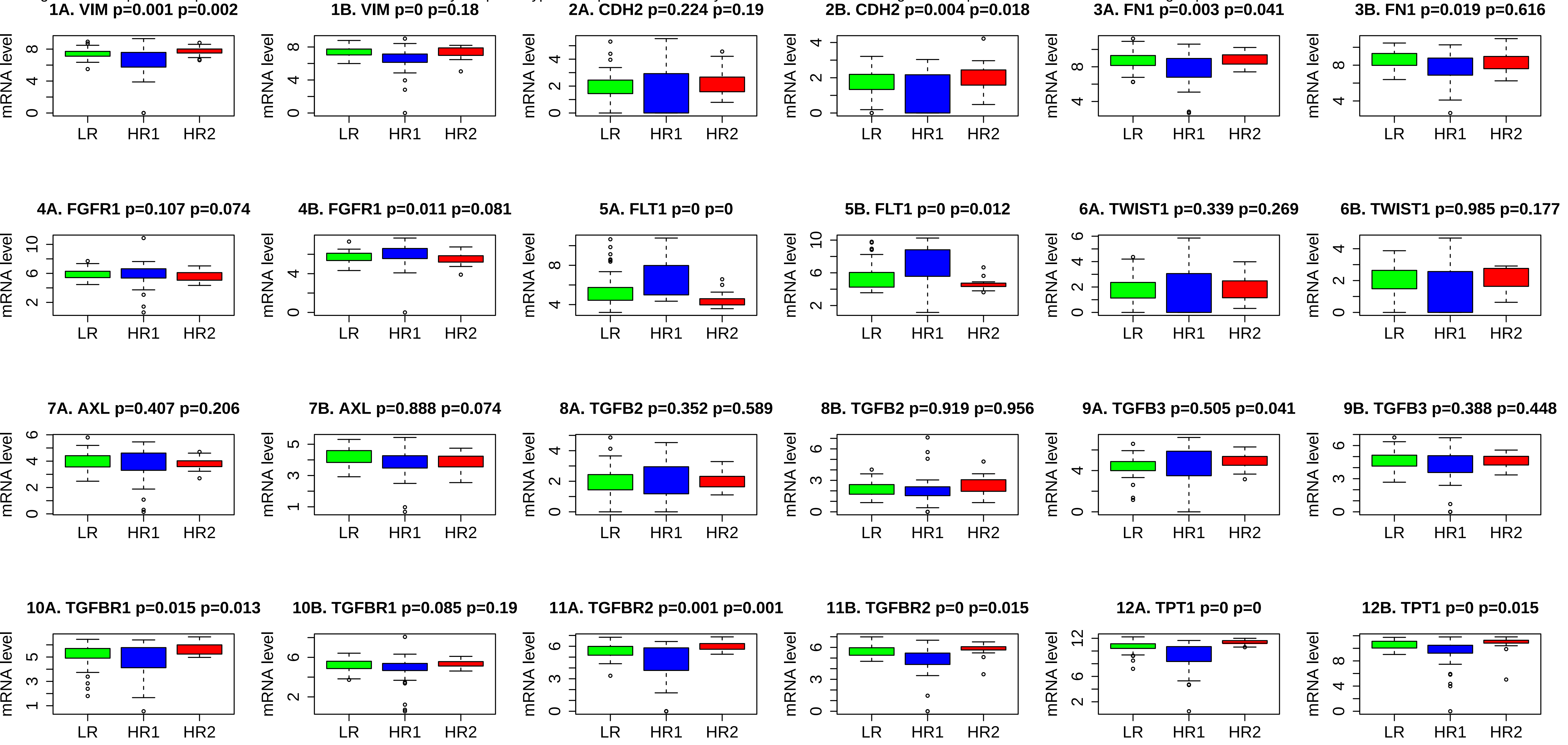

Figure S3. Boxplots of expression levels of markers of epithelial phenotype over 222 stage II CRC patients divided into three subgroups

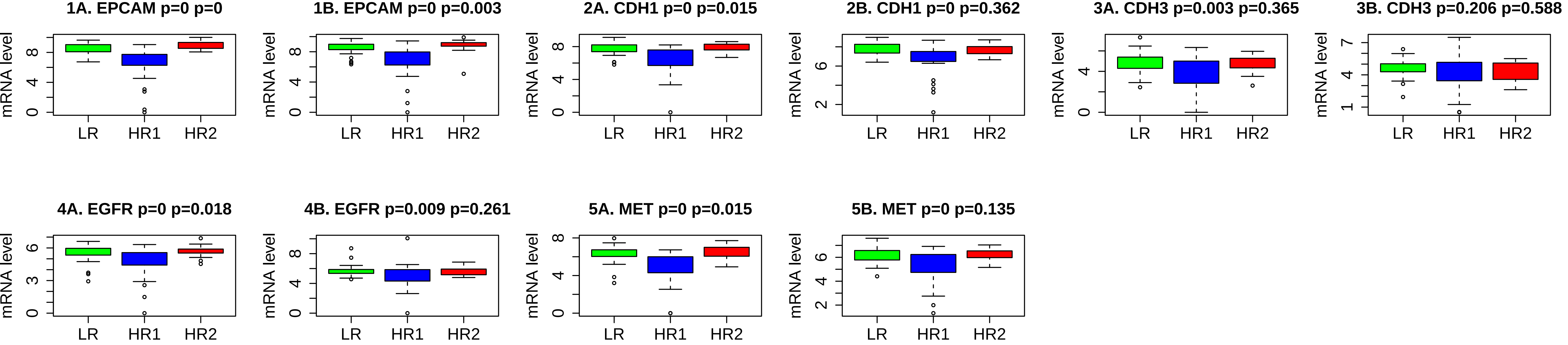

Figure S4. Boxplots of expression levels of markers of 14 common tumor infiltrating immune cells over 222 stage II CRC patients divided into three subgroups

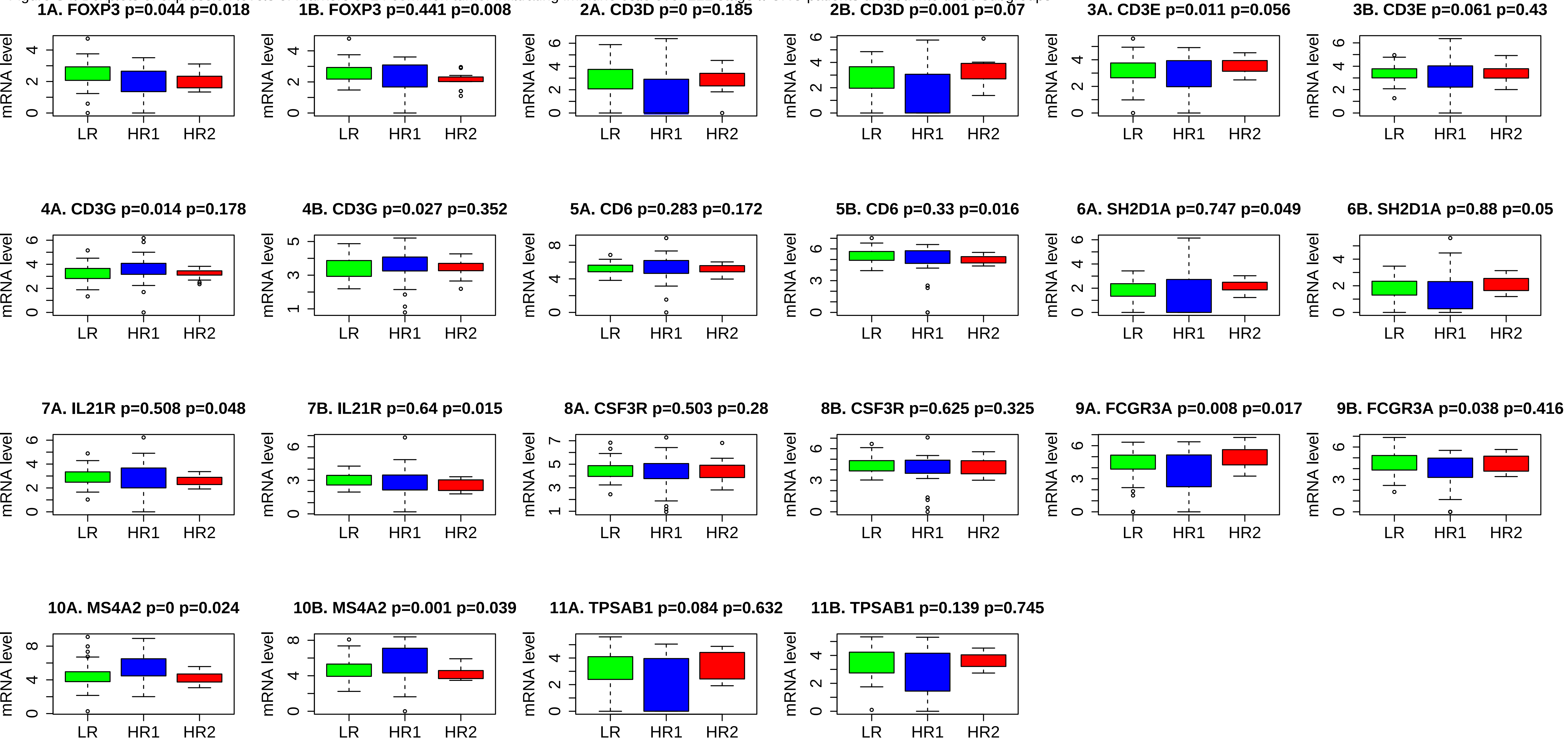

Figure S5. Boxplots of expression levels of markers of 14 common tumor infiltrating immune cells over 222 stage II CRC patients divided into three subgroups

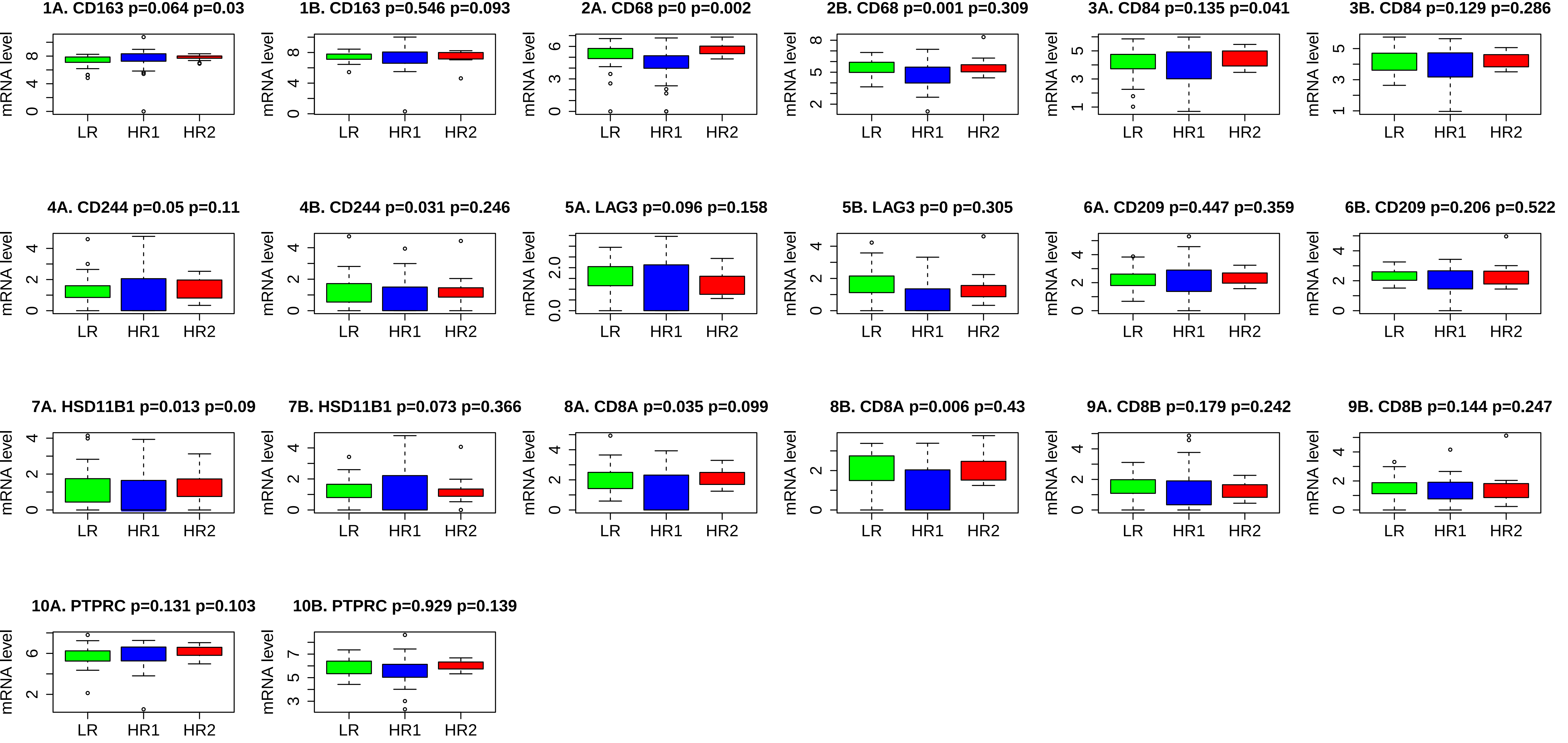

Figure S6. Boxplots of expression levels of markers of 14 common tumor infiltrating immune cells over 222 stage II CRC patients divided into three subgroups

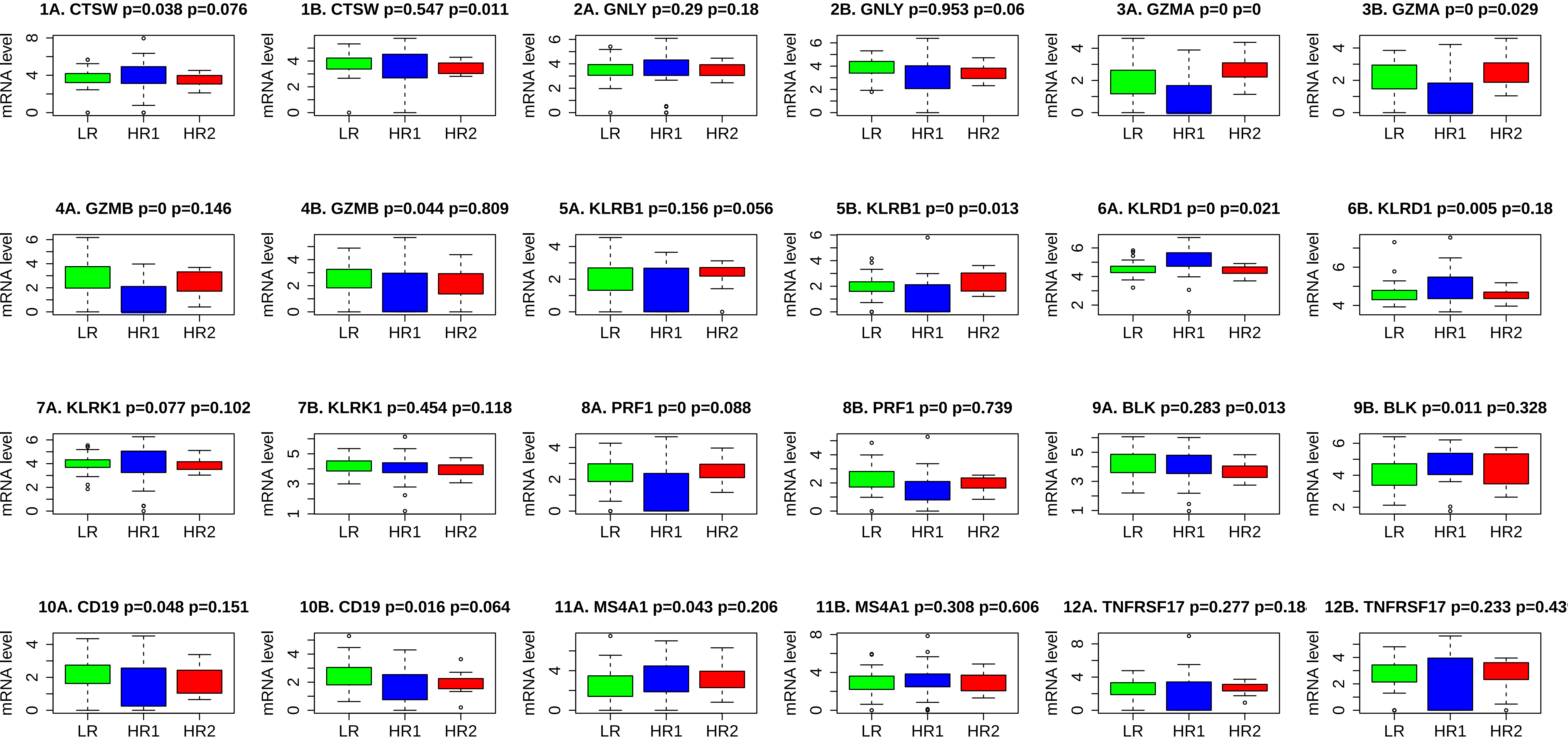

Figure S7. Boxplots of expression levels of markers of hypoxia phenotype over 222 stage II CRC patients divided into three subgroups

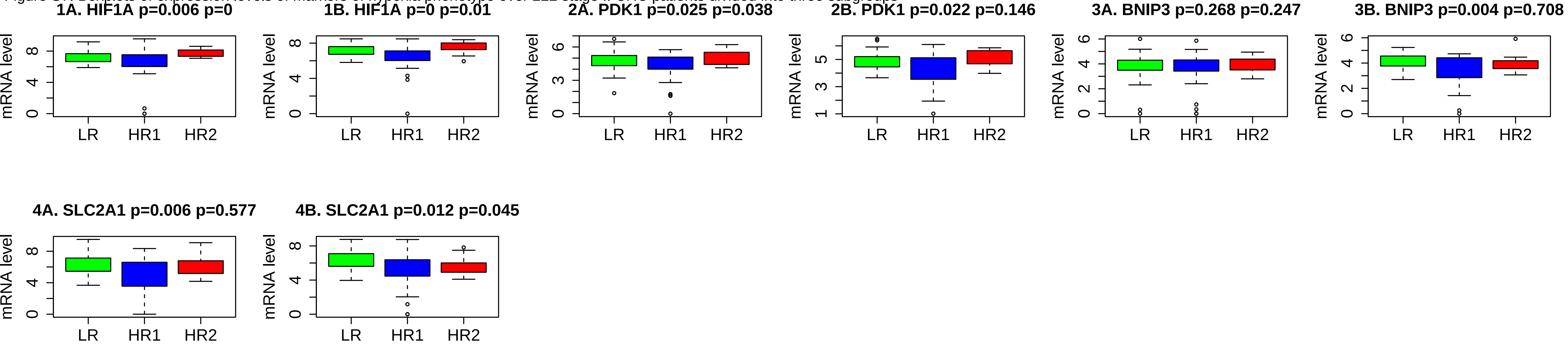

Supplement: Supplementary file 1 [file DataSheet_1.pdf]
