# Supplementary material for: Dissected subgroups predict the risk of recurrence of stage II colorectal cancer and select rational treatment
Source: Front Immunol. 2023 Mar 23;14:1103741. doi: 10.3389/fimmu.2023.1103741 (PMC10076777; doi:10.3389/fimmu.2023.1103741)
Supplement: Supplementary file 2 [file Table_1.doc]

Supplementary Table S1. List of genes in the signature of TFunctionalProg high-risk subgroup 1

| **Genename** | **StatisticsOccurenceInCrossvalidation** | **RegulationDir_Reccurence(Highrisk)Group** |
| --- | --- | --- |
| ATG101 | 200 | Down |
| ATP5ME | 169 | Down |
| CACYBP | 177 | Down |
| CAV2 | 190 | Up |
| CCDC137 | 187 | Down |
| CDK4 | 167 | Down |
| CKAP4 | 194 | Down |
| CLPP | 183 | Down |
| COA6 | 178 | Down |
| DCXR | 185 | Down |
| DUSP23 | 200 | Down |
| E2F8 | 194 | Down |
| EIF3I | 178 | Down |
| ELOB | 194 | Down |
| ENO1 | 199 | Down |
| EPHB2 | 200 | Down |
| FARSA | 200 | Down |
| HIF3A | 200 | Up |
| HIST1H2AH | 182 | Down |
| HIST1H2BK | 165 | Down |
| IQANK1 | 172 | Down |
| MCM2 | 200 | Down |
| MCM6 | 192 | Down |
| MTA2 | 198 | Down |
| NR2C2AP | 172 | Down |
| PCNA | 198 | Down |
| PIR | 188 | Down |
| PLIN3 | 194 | Down |
| PRDX2 | 167 | Down |
| ROMO1 | 196 | Down |
| RPA2 | 162 | Down |
| SETD1A | 176 | Down |
| SMPD2 | 197 | Down |
| SNAPC4 | 185 | Down |
| SPATA6 | 197 | Up |
| ST13 | 182 | Down |
| THAP4 | 192 | Down |
| TNFAIP3 | 185 | Down |
| TP73-AS1 | 200 | Up |
| UTP18 | 174 | Down |
| WT1 | 186 | Up |

Supplementary Table S2. List of genes in the signature of TFunctionalProg high-risk subgroup 2

| Genename | StatisticsOccurenceInCrossvalidation | RegulationDir_Reccurence(Highrisk)Group |
| --- | --- | --- |
| APLP2 | 181 | Up |
| BTF3 | 198 | Up |
| CAB39 | 177 | Up |
| COPB2 | 176 | Up |
| CRTAP | 200 | Up |
| CSF1 | 200 | Down |
| CSNK1A1 | 168 | Up |
| DSG2 | 188 | Up |
| EIF1AY | 192 | Up |
| ENDOD1 | 200 | Up |
| FCHO2 | 200 | Up |
| FRRS1 | 161 | Up |
| FUT6 | 166 | Down |
| HIST1H2AC | 200 | Up |
| IGFLR1 | 182 | Down |
| ITM2B | 181 | Up |
| MEDAG | 164 | Up |
| MYL12A | 200 | Up |
| MYL12B | 200 | Up |
| OCLN | 191 | Up |
| PAK1 | 169 | Up |
| PFDN5 | 168 | Up |
| PHLDB2 | 163 | Up |
| PMS2P4 | 198 | Up |
| RAB27B | 181 | Up |
| RALB | 200 | Up |
| RALBP1 | 177 | Up |
| RHOA | 194 | Up |
| RSF1 | 180 | Up |
| SEL1L | 195 | Up |
| SPON1 | 188 | Up |
| SPPL3 | 178 | Up |
| TM9SF2 | 184 | Up |
| TMEM30B | 200 | Up |
| TPT1 | 164 | Up |
| TRIM65 | 194 | Down |
| TXNDC17 | 176 | Up |
| VAPA | 196 | Up |
| YWHAE | 200 | Up |
| ZNF74 | 192 | Down |

Supplementary Table S3. Enriched biological pathways of genes in the signature of TFunctionalProg high-risk subgroup 2.

| **Biological pathway** | **No. of genes in the dataset** | **Percentage of genes** | **P-value (Hypergeometric test)** |
| --- | --- | --- | --- |
| Regulation of cytoplasmic and nuclear SMAD2/3 signaling | 6 | 35,29411765 | 9,72494E-05 |
| TGF-beta receptor signaling | 6 | 35,29411765 | 9,72494E-05 |
| Regulation of nuclear SMAD2/3 signaling | 6 | 35,29411765 | 9,72494E-05 |
| ALK1 signaling events | 6 | 35,29411765 | 0,000129245 |
| ALK1 pathway | 6 | 35,29411765 | 0,000136088 |
| Alpha6Beta4Integrin | 3 | 17,64705882 | 0,000263138 |
| FoxO family signaling | 3 | 17,64705882 | 0,000279831 |
| Apoptotic cleavage of cell adhesion proteins | 2 | 11,76470588 | 0,000446654 |
| Semaphorin interactions | 3 | 17,64705882 | 0,000617129 |
| Regulation of p38-alpha and p38-beta | 4 | 23,52941176 | 0,00081309 |
| EPHA2 forward signaling | 2 | 11,76470588 | 0,001144431 |
| p38 MAPK signaling pathway | 4 | 23,52941176 | 0,001382216 |
| Sema4D induced cell migration and growth-cone collapse | 2 | 11,76470588 | 0,001538632 |
| S1P2 pathway | 2 | 11,76470588 | 0,002151029 |
| CDC42 signaling events | 7 | 41,17647059 | 0,002289032 |
| Sema4D in semaphorin signaling | 2 | 11,76470588 | 0,002319424 |
| PLK1 signaling events | 3 | 17,64705882 | 0,002522717 |
| Regulation of CDC42 activity | 7 | 41,17647059 | 0,002530136 |
| BMP receptor signaling | 4 | 23,52941176 | 0,002670031 |
| Polo-like kinase signaling events in the cell cycle | 3 | 17,64705882 | 0,002884017 |
| IL1-mediated signaling events | 4 | 23,52941176 | 0,003030502 |
| Lissencephaly gene (LIS1) in neuronal migration and development | 2 | 11,76470588 | 0,003053283 |
| Netrin-mediated signaling events | 2 | 11,76470588 | 0,003251668 |
| Signal transduction by L1 | 2 | 11,76470588 | 0,003882157 |
| AP-1 transcription factor network | 6 | 35,29411765 | 0,004317945 |
| p75 NTR receptor-mediated signalling | 2 | 11,76470588 | 0,004804158 |
| Apoptotic cleavage of cellular proteins | 2 | 11,76470588 | 0,004804158 |
| Integrin-linked kinase signaling | 6 | 35,29411765 | 0,005594035 |
| EphrinA-EPHA pathway | 2 | 11,76470588 | 0,005817424 |
| Apoptotic execution phase | 2 | 11,76470588 | 0,007209553 |
| TNF receptor signaling pathway | 4 | 23,52941176 | 0,007283198 |
| Signaling events mediated by PTP1B | 2 | 11,76470588 | 0,008421488 |
